# Supplementary material for: Transcriptional Integration of Meiotic Prophase I Progression and Early Oocyte Differentiation
Source: bioRxiv. 2025 Jan 6:2025.01.06.631470. Preprint. [Version 1] doi: 10.1101/2025.01.06.631470 (PMC11741336; doi:10.1101/2025.01.06.631470)
Supplement: 1 [file NIHPP2025.01.06.631470V1-supplement-1.pdf]

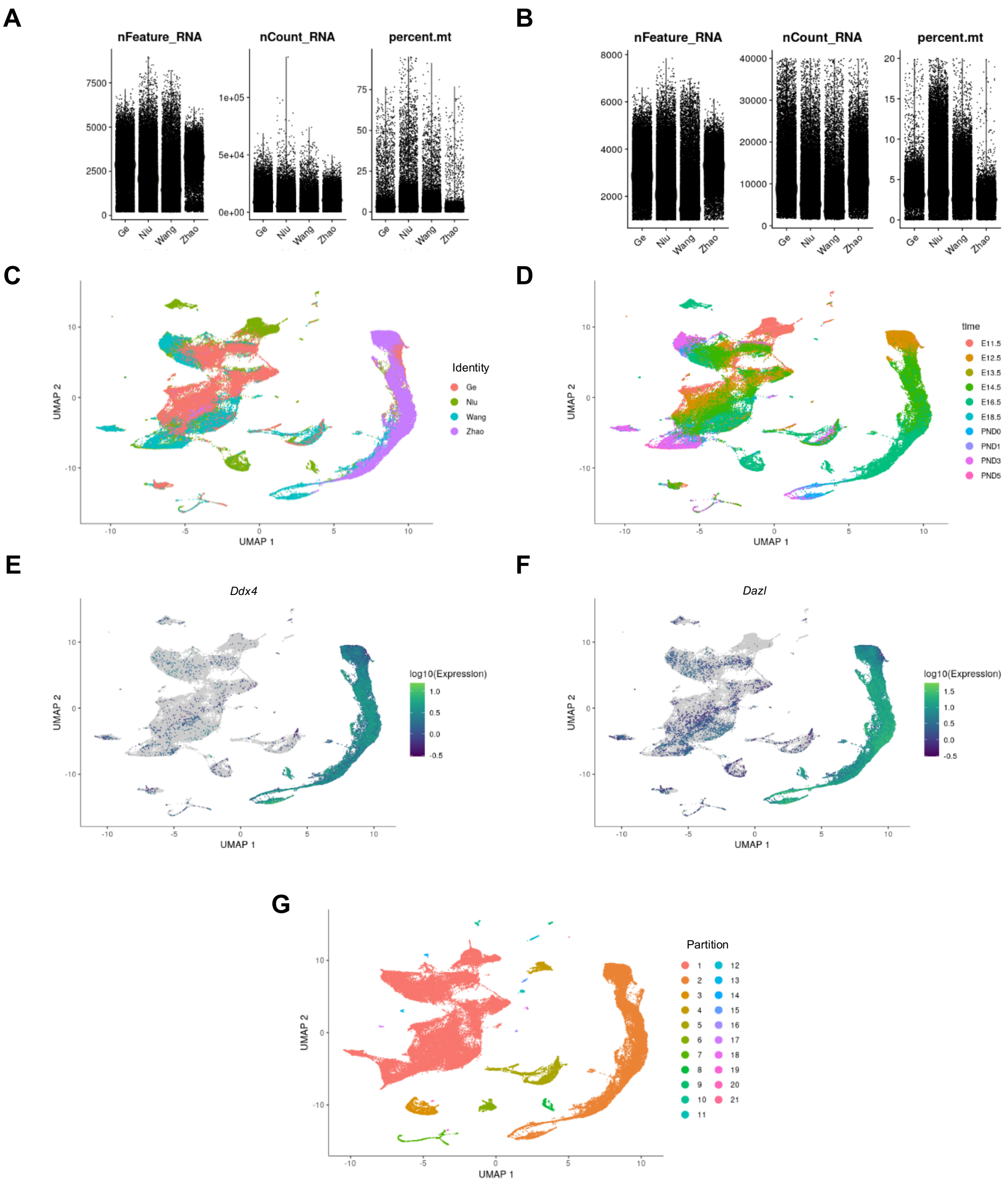

Figure S1 – Overview scRNA-seq dataset integration. Scattered boxplots showing the QC metric distributions of the four individual datasets before (A) and after (B) setting thresholds for number of features (nFeature), number of counts (nCount), and percent mitochondrial content (percent.mt). UMAP of all cells that passed QC colored by dataset origin (C) timepoint (D) log10 expression of germ cell markers *Ddx4* (E) or *Dazl* (F) and unbiased partition assignment (G).

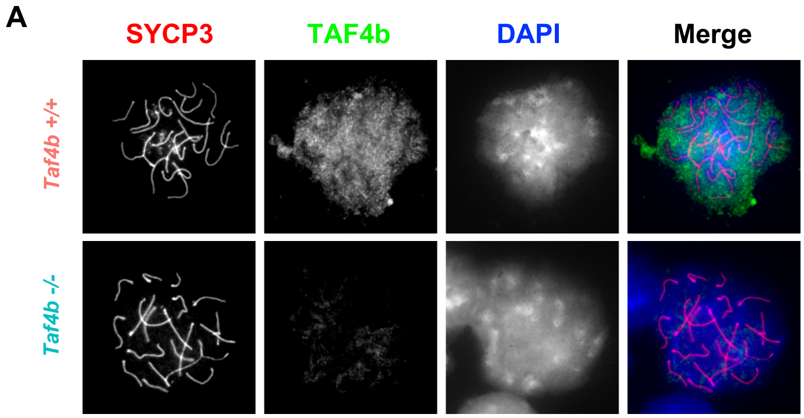

Figure S2 – Validation of TAF4b antibody use in immunofluorescence. (A) Images of pachytene chromatin spreads from *Taf4b*+/+ (top) and *Taf4b*-/- (bottom) E18.5 ovaries stained for TAF4b (green), SYCP3 (red) and DAPI (blue).

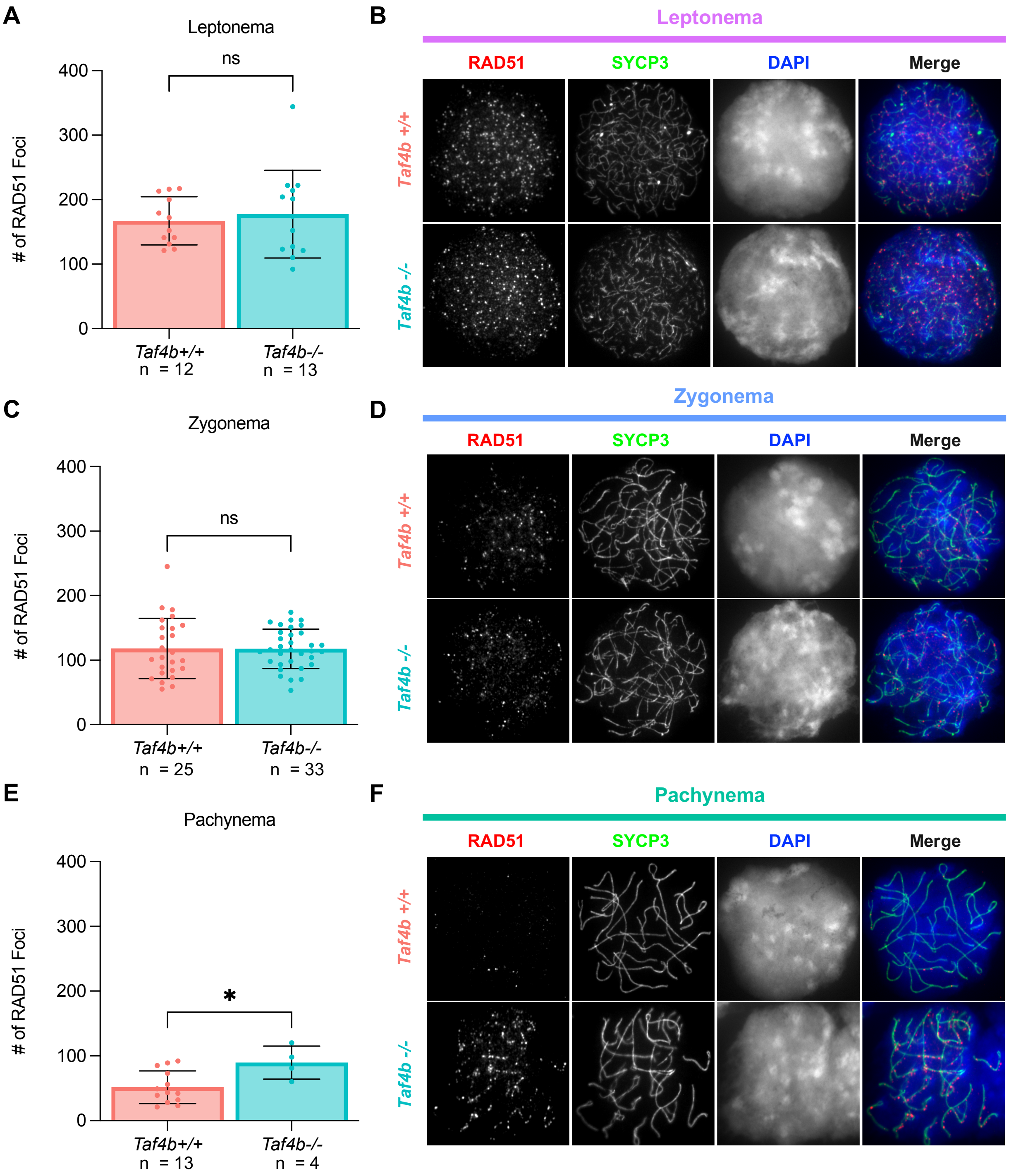

Figure S3 – *Taf4b-/-* oocytes have elevated levels of RAD51 foci during pachynema. Quantification of RAD51 foci in chromatin spreads during leptonema (A) zygonema (C) and pachynema (E). Spreads were pooled from two E16.5 mice per genotype, n = the number of spreads analyzed. Images of spreads stained with RAD51 (red) SYCP3 (green) and DAPI (blue) from each genotype during leptonema (B) zygonema (D) and

pachynema (F). Dots represent individual spreads, bar height represents the sample mean, and error bars represent standard error of the mean. Statistical significance for stage specific comparisons was determined using a two-tailed T-test, ns=not significant, and * $p < 0.05$.

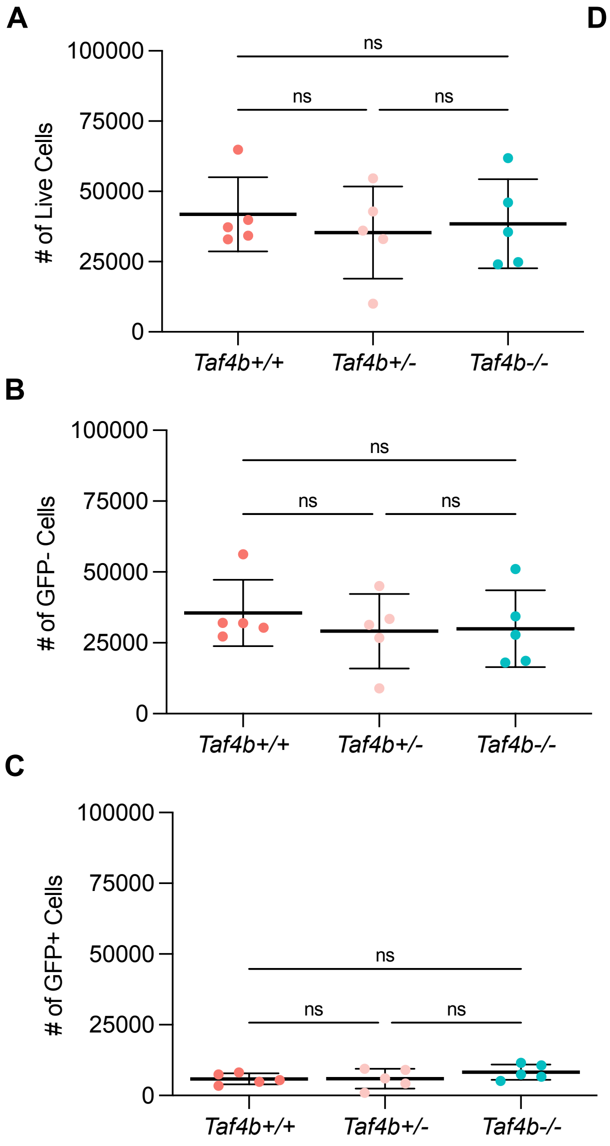

D

Genotype	Sample	# of GFP+ Cells
Taf4b+/+	1	9839
Taf4b+/+	2	4921
Taf4b+/+	3	4672
Taf4b+/+	4	3312
Taf4b+/+	5	5973
Taf4b+/-	1	4181
Taf4b+/-	2	2712
Taf4b+/-	3	6046
Taf4b+/-	4	8721
Taf4b+/-	5	8331
Taf4b-/-	1	7098
Taf4b-/-	2	6250
Taf4b-/-	3	5134
Taf4b-/-	4	10859
Taf4b-/-	5	11166

Figure S4 – FACS summary of E18.5 samples sent for bulk RNA-seq. Charts depicting the number of live (A) GFP$^-$ (B) and GFP$^+$ (C) cells collected per sample during FACS of E18.5 *Taf4b+/+, +/-*, or *-/-*;Oct4-EGFP ovaries. Dots represent an individual sample, bars represent sample mean, and error bars represent standard deviation. Statistical significance was determined using an ordinary one-way ANOVA with multiple comparisons, ns = not significant. (D) Table of GFP$^+$ cell numbers per sample submitted for bulk RNA-sequencing with Azenta/Genewiz.

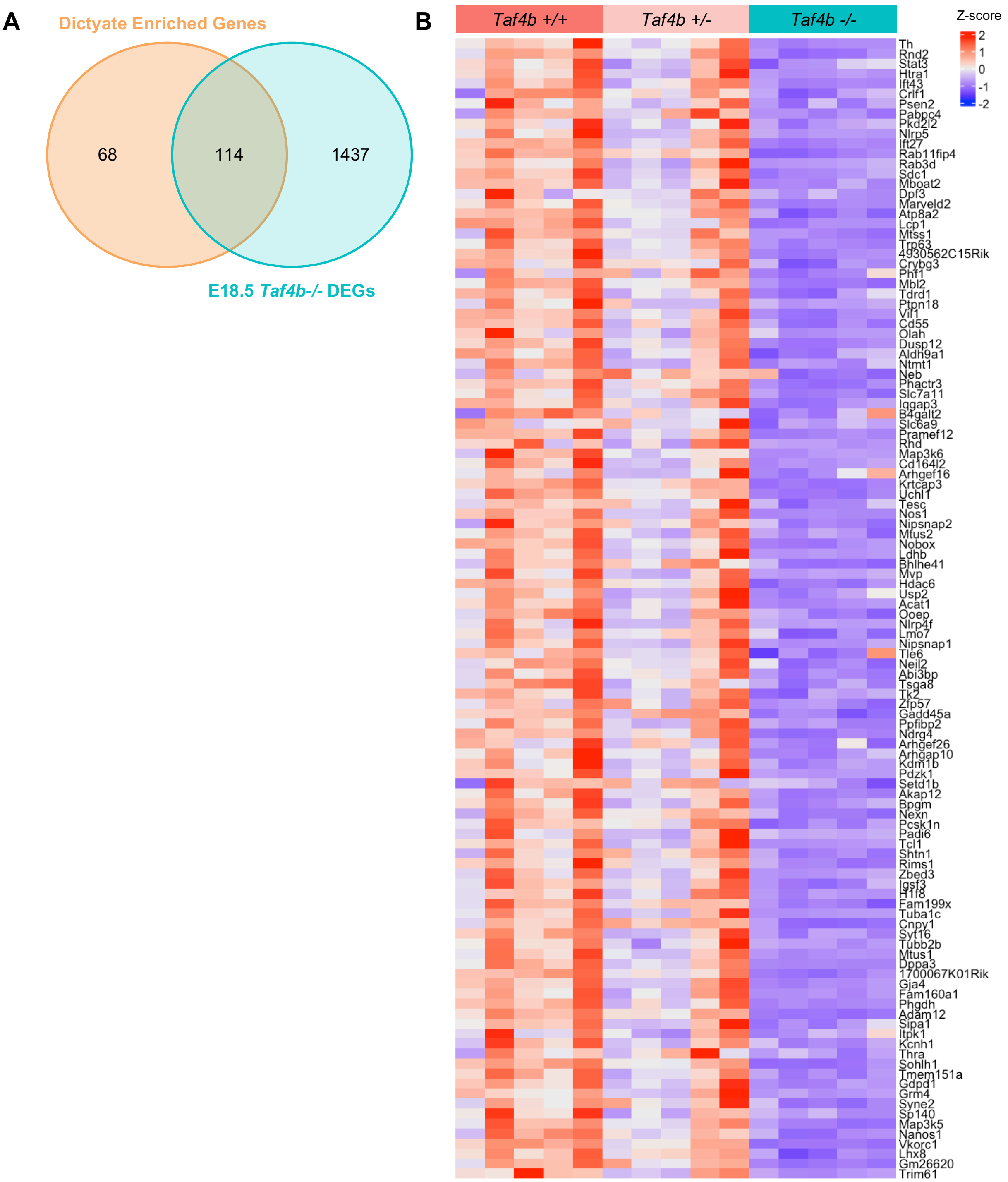

Figure S5 – Reduced expression of dictyate enriched genes in E18.5 *Taf4b-/-* oocytes. (A) Venn diagram of E18.5 *Taf4b-/-* DEG list compared with dictyate enriched gene list (Niu & Spradling, 2022). (B) Heatmap of 114 dictyate enriched genes that are also differentially expressed in E18.5 *Taf4b-/-* oocytes.

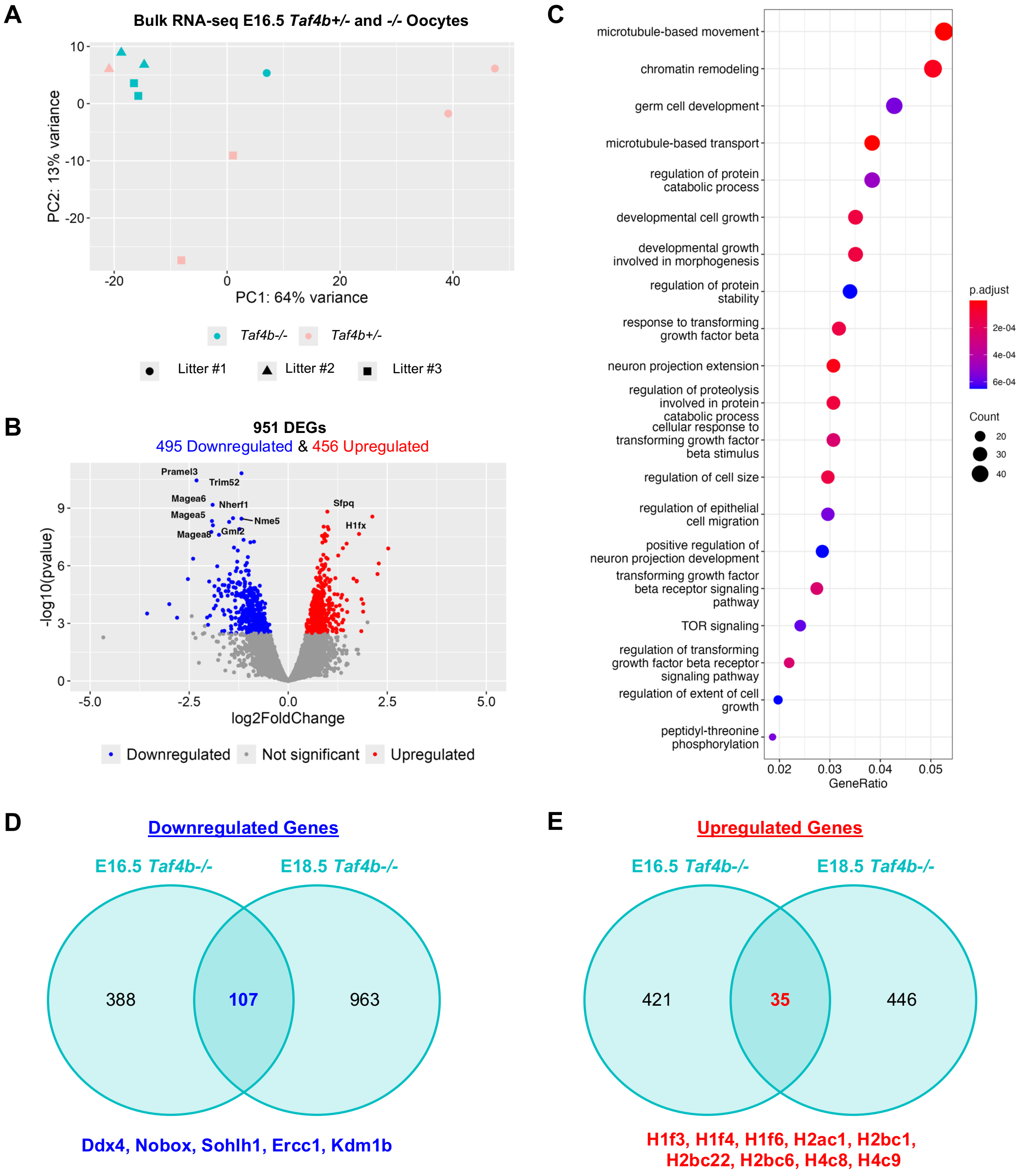

Figure S6 – Comparison of *Taf4b-/-* DEGs at E16.5 and E18.5. (A) PCA plot of E16.5 samples labelled based on genotype and collection date. (B) Volcano plot of DEGs (protein-coding, padj <0.05, avg TPM > 1) with top 10 most significant labelled. (C) Dotplot of GO biological process analysis of 951 DEGs. Venn diagram of genes that were downregulated (D) or upregulated (E) *Taf4b-/-* oocytes at E16.5 and E18.5.

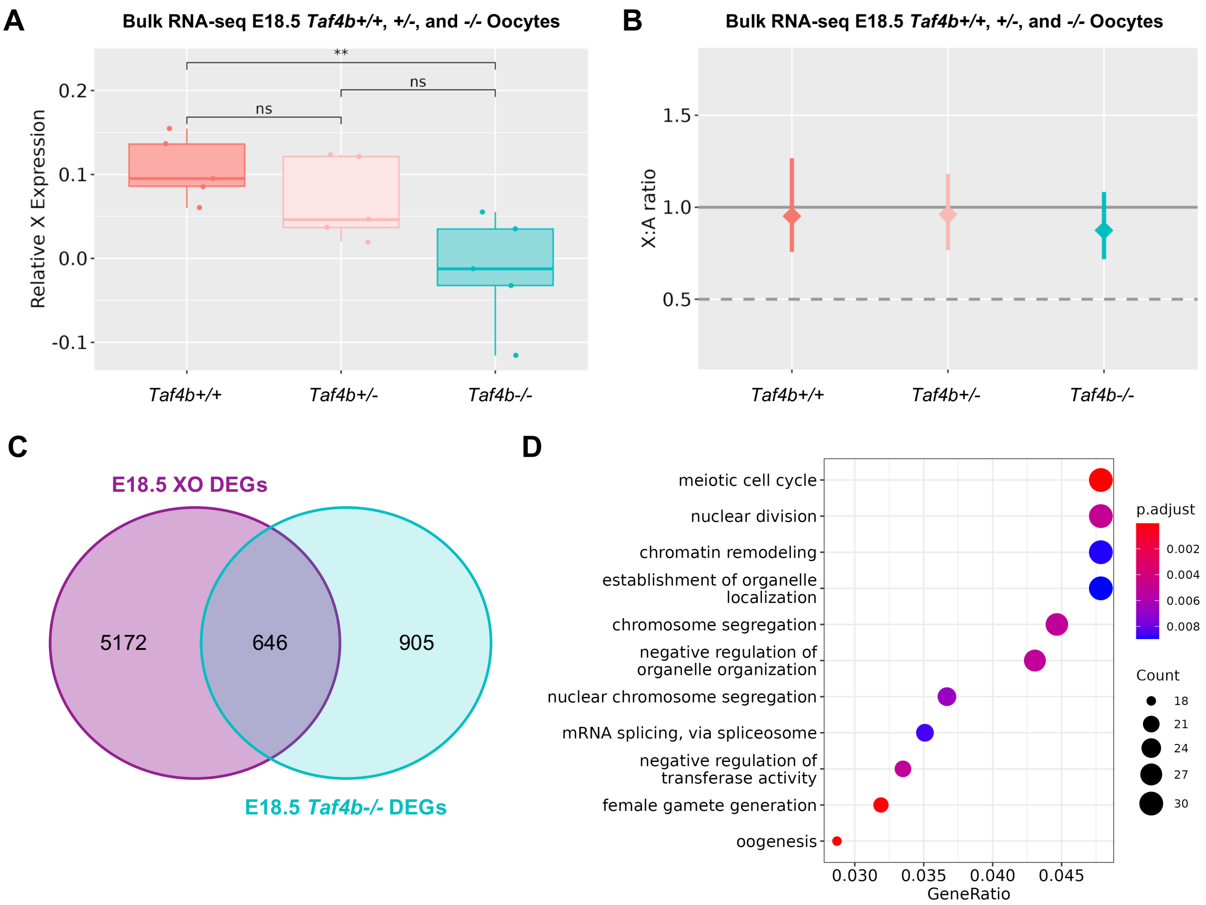

Figure S7 – Reduced X chromosome expression in E18.5 *Taf4b-/-* oocytes. (A) Box plots of relative X expression (RXE) calculations after filtering for avg TPM > 1 and adding pseudocounts for log transformation of *Taf4b*+/+, +/-, and -/- samples. Statistical significance was determined using Welch's T-test, ** p <0.01. Boxplot shows interquartile range from 25th to 75th percentile and median (solid line), whiskers represent the minimum and maximum values, and dots represent individual samples. (B) X:A ratio plot comparing *Taf4b*+/+, +/-, and -/- samples based on pairwise CI calculations performed after filtering for avg TPM > 1. Solid gray line represents full dosage compensation between X chromosomes and autosomes. Dashed gray line represents half dosage compensation of the X chromosome with the autosomes. Median as well as upper and lowers confidence intervals are plotted. (C) Venn diagram of E18.5 *Taf4b-/-* DEG list compared with E18.5 XO DEGs (protein-coding, padj <0.05, avg TPM > 1) published previously (Gura et al., 2022) (D) Dotplot of GO biological process analysis of the 642 DEGs shared between E18.5 *Taf4b-/-* and E18.5 XO oocytes.
